# Current trends and biases in groundwater modelling using the community-driven groundwater model portal (GroMoPo)

**DOI:** 10.1007/s10040-025-02882-7

**Published:** 2025-03-28

**Authors:** Daniel Zamrsky, Sacha Ruzzante, Kyle Compare, Daniel Kretschmer, Sam Zipper, Kevin M. Befus, Robert Reinecke, Yara Pasner, Tom Gleeson, Kristen Jordan, Mark Cuthbert, Anthony M. Castronova, Thorsten Wagener, Marc F. P. Bierkens

**Affiliations:** 1 Department of Physical Geography, Utrecht University, Utrecht, The Netherlands; 2 Department of Civil Engineering and School of Earth and Ocean Sciences, University of Victoria, Victoria, Canada; 3 Department of Earth, Ocean and Atmospheric Science, Florida State University, Tallahassee, FL USA; 4 Institute of Environmental Science and Geography, University of Potsdam, Potsdam, Germany; 5 Institute of Geography, Johannes Gutenberg-University Mainz, Mainz, Germany; 6 Kansas Geological Survey, University of Kansas, Lawrence, KS USA; 7 Department of Geology, University of Kansas, Lawrence, KS USA; 8 Department of Geosciences, University of Arkansas, Fayetteville, AR USA; 9 University of California, Davis, USA; 10 School of Earth and Environmental Sciences, Cardiff University, Cardiff, UK; 11 Consortium of Universities for the Advancement of Hydrologic Sciences, Inc, Arlington, USA; 12 Deltares, Unit Subsurface and Groundwater Systems, Utrecht, The Netherlands

**Keywords:** Groundwater models, History of hydrogeology, Groundwater statistics, Community, Database

## Abstract

Groundwater, Earth’s largest nonfrozen freshwater reservoir, is vital for water supply security. Groundwater models help to manage complex domestic, agricultural, and industrial water demands while preserving ecosystem health under climate change. The community-driven groundwater model portal (GroMoPo) hosts groundwater model metadata to analyse biases and distribution of groundwater models. Over 450 models are currently featured on GroMoPo, with most models from high-GDP countries at local-to-regional scales. The GroMoPo initiative addresses current knowledge gaps and facilitates future collaboration and data sharing.

**Supplementary Information:**

The online version contains supplementary material available at 10.1007/s10040-025-02882-7

## Résumé

Les eaux souterraines, le plus grand réservoir d’eau douce non gelée de la planète, sont essentielles à la sécurité de l’approvisionnement en eau. Les modèles d’eaux souterraines aident à gérer les demandes complexes en eau domestique, agricole et industrielle tout en préservant la santé des écosystèmes face au changement climatique. Le portail communautaire de modèles d’eaux souterraines (GroMoPo) héberge des métadonnées de modèles d’eaux souterraines pour analyser les biais et la distribution des modèles d’eaux souterraines. Plus de 450 modèles sont actuellement présentés sur GroMoPo, la plupart provenant de pays à PIB élevé, à l’échelle locale ou régionale. L’initiative GroMoPo comble les lacunes actuelles en matière de connaissances et facilite la collaboration et le partage de données futurs.

## Resumen

La mayor reserva de agua dulce no congelada de la Tierra, el agua subterránea, es vital para la seguridad del abastecimiento de agua. Los modelos de aguas subterráneas ayudan a gestionar la compleja demanda de agua para uso doméstico, agrícola e industrial, preservando al mismo tiempo la salud de los ecosistemas en un contexto de cambio climático. El portal de modelos de aguas subterráneas orientado a la comunidad (GroMoPo) contiene metadatos de modelos de aguas subterráneas para analizar los sesgos y la distribución de los modelos. En la actualidad, GroMoPo incluye más de 450 modelos, la mayoría de los cuales proceden de países con un PIB elevado y se elaboran a escala local y regional. La iniciativa GroMoPo aborda las actuales lagunas de conocimiento y facilita la colaboración y el intercambio de datos en el futuro.

## Abstract (zh)

地下水是地球上最大的非冰冻淡水储备，对于水供应安全至关重要。地下水模型有助于管理复杂的家庭、农业和工业用水需求，同时在气候变化的背景下保护生态系统健康。社区驱动的地下水模型门户（GroMoPo）汇集了地下水模型的元数据，以分析地下水模型的偏差和分布。目前，GroMoPo上展示了超过450个模型，绝大多数模型来自高GDP国家，涵盖从地方到区域的规模。GroMoPo倡议旨在填补当前的知识空白，促进未来的合作与数据共享。

## Resumo

As águas subterrâneas são os maiores reservatórios de água doce não congelada da Terra, e são essenciais para a garantia de segurança hídrica. Os modelos de águas subterrâneas auxiliam não só no gerenciamento de complexas demandas hídricas domésticas, agrícolas e industriais, como também na preservação dos ecossistemas frente às mudanças climáticas. O portal colaborativo de modelagem de águas subterrâneas (GroMoPo) armazena metadados diversos modelos hidrogeológicos para analisar os vieses e as distribuições desses modelos. Atualmente, mais de 450 modelos estão reunidos no GroMoPo, sendo a maioria proveniente de países de alto PIB e em escala local a regional. A iniciativa GroMoPo busca preencher lacunas de conhecimento e promover a colaboração e o compartilhamento de dados no futuro.

## Introduction

More than 99% of Earth’s liquid freshwater is stored underground, making groundwater an essential freshwater resource worldwide (UN Water 2022). Globally, many communities, especially in rural environments, rely solely on groundwater as their drinking water supply (UN Water 2022). Additionally, many densely populated regions rely on groundwater for industrial and agricultural production (Lall et al. 2020). This dependency on groundwater resources is increasing over time and has already led to groundwater depletion in many areas, while many others are under threat (Bierkens and Wada 2019; Jasechko and Perrone 2021). In parallel, the number of local-to-regional-scale groundwater studies that focus on groundwater modelling has steadily increased over the past 40 years (Jia et al. 2020). This trend is likely to continue since groundwater models have become a commonly used tool across the hydrogeological community, partly driven by the proliferation of groundwater modelling codes, software packages, and advances in computing power. These models provide better understanding of spatiotemporal patterns of groundwater flow and quality and interactions between groundwater and surface water. Furthermore, these models improve the fundamental mechanistic understanding of hydrologic systems, which can then be applied to real-time or forecasting operational decision support tools. However, this increasing number of groundwater models is accompanied by significant challenges.

Every groundwater model can provide valuable insights for local decision-makers and the wider groundwater community. The choices made while building a groundwater model include components that are subjective and driven by regional experience or schools of thought. This local knowledge comes into play because building a groundwater model involves simulating key processes that influence groundwater flow, as well as combining multiple input datasets, defining boundary conditions, and model validation with local observational data (Wagener et al. 2021). These choices and knowledge, which inform the hydrogeological conceptual model of the study area, are then embedded in the numerical implementation of the groundwater models and can also provide valuable insights for the wider groundwater modelling community. Yet local- and regional-scale groundwater models generally reach a small audience, and the knowledge contained within these studies is not efficiently shared across the groundwater modelling community. In contrast, the recent development of global groundwater models (de Graaf et al. 2017; Gleeson et al. 2021; Verkaik et al. 2024) has brought more attention to groundwater issues worldwide such as depletion and pollution (Lall et al. 2020). The missing link between global groundwater models and local-to-regional-scale observations and model simulations has been identified as a limiting factor for the evaluation and improvement of these global groundwater models (de Graaf et al. 2017; Gleeson et al. 2021; Verkaik et al. 2024).

Biases in the geographical distribution of environmental studies have long been identified in environmental sciences. As early as 1896, the editors of the* Botanical Gazette* noted that botanical science “rests largely upon the results of researches carried on in the north temperate zone … situated between the parallels of 40 and 55” (Editorials 1896). More recently, it has been noted that biases towards protected areas, the temperate zone, and wealthy countries potentially “limit the global relevance of ecological research” (Martin et al. 2012). Hydrological research similarly tends to concentrate in wealthier countries and the temperate climate zone (Addor and Melsen 2019; Burt and McDonnell 2015), something already reflected in highly biased observational networks, e.g. streamflow gauging stations (Krabbenhoft et al. 2022). Studies on hydrological hazards, such as floods and droughts, show a strong bias towards wealthy regions, often in strong contrast to the most impacted regions because of such extremes have on society (Stein et al. 2024). Partly, this bias is likely related to researchers from wealthier countries being more likely to afford the publishing fees in peer-reviewed journals, while there might be a large amount of grey literature and studies on regional groundwater models in less economically developed regions. Furthermore, this bias can be potentially linked to limited local observations, training and teaching capacities and access to computational facilities. Although the groundwater modelling community has been slow to address this issue, the time has come to confront its shortcomings. Under-represented regions serve as blind spots that impede the ability to understand the present and prepare for the future in an increasingly interconnected world (Wilby 2019). A related challenge is the under-representation of researchers from marginalised regions and communities. Local capacity-building is an often-cited strategy to address both challenges (Scheihing et al. 2022), but too often high-impact research excludes local researchers (Minasny et al. 2020; North et al. 2020; Tavernier et al. 2023).

Sharing the knowledge created through building, running, and evaluating groundwater models is not yet common within the groundwater community, which can be attributed to multiple related reasons. Many groundwater models are built with proprietary software (Zipper et al. 2022) and/or contain private input data and information (Zipper et al. 2019). In such cases, these groundwater models cannot be made open source and fully accessible. Furthermore, groundwater models are often developed for site-specific investigations and are rarely published in peer-reviewed journals. Increasing the visibility and discoverability of such groundwater models may increase the chances of reusability and collaboration while safeguarding sensitive data.

To address spatial bias in published models and promote access to local-to-regional-scale models, this study promotes that groundwater models (and publications thereof) need to incorporate the FAIR (findable, accessible, interoperable, and reusable) principle (Stall et al. 2017). FAIR groundwater models can better accommodate research that spans model scales, enables more efficient and accessible knowledge exchange within the groundwater modelling community, and avoids duplication of efforts (Reinecke et al. 2022). FAIR and open-source data are becoming a community standard and are often required for publications and grants by journals and funding agencies (Reinecke et al. 2022). Promoting the FAIR principle within the groundwater modelling community will contribute to changing the current incentive structures in the academic system that can hinder common-good activities such as model and data sharing (Verbeke 2023). Sharing groundwater model input data and results is becoming easier with a growing number of freely accessible storage services online (such as HydroShare, Zenodo, or PANGAEA). Therefore, establishing community standards for data sharing is crucial at this stage and can help groundwater modellers share their models and research. In recent years, the groundwater modelling field has undergone a steady growth in publication rate (Jia et al. 2020). This can metaphorically increase the number of needles in an ever-growing haystack of models (Stein et al. 2022).

## GroMoPo goals and overview

**Fig. 1:**
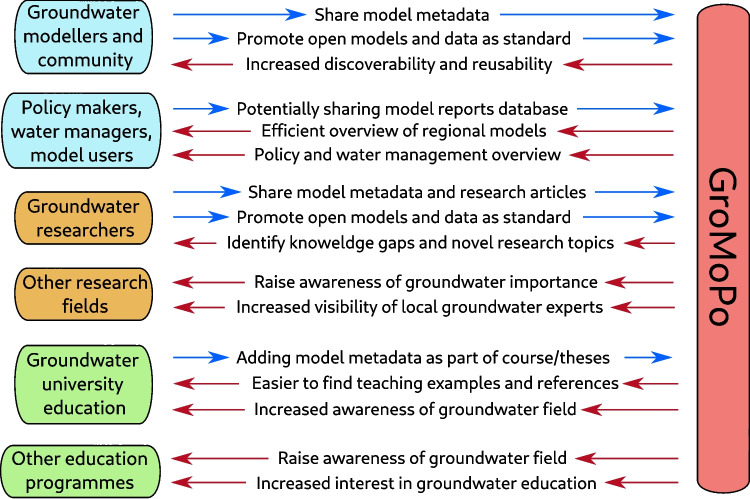
Envisioned GroMoPo contributions and potential input from (blue arrows) and benefits to (red arrows) groundwater community and beyond (modified from Zipper et al. 2023)

A community-driven initiative called Groundwater Model Portal (GroMoPo) was initiated to help promote the adoption of FAIR protocol in groundwater modelling by collecting and distributing groundwater models via an open-source online platform (Zipper et al. 2023). GroMoPo is not solely a platform for sharing software and modelling code such as previous attempts to create global hydrological or hydrogeological databases (Wagener et al. 2004). Instead, GroMoPo provides an information-sharing point for the wider groundwater modelling community where documentation about model setup, procedures, and goals is stored and shared to hopefully attract a wider audience and help promote uniform standards and practices in groundwater modelling. Groundwater modellers, managers, policy-makers, researchers, and educators can all potentially benefit from GroMoPo by making groundwater models and research more accessible to the wider public (Fig. 1). GroMoPo can help connect model builders and model users throughout the global groundwater community. GroMoPo can also serve as an educational tool through direct integration into the course material and by providing example models for educators and students worldwide. Furthermore, GroMoPo may provide a gateway into groundwater modelling for other scientific fields and lead to increased interdisciplinary research in future work.

The GroMoPo database is created by manually extracting metadata from over 450 groundwater model reports and articles (as of December 2024). Initially, the metadata were gathered from a dataset of approximately 150 studies collected by the GroMoPo team. This dataset was expanded further through a systematic search using the Web of Science search engine. For more details, refer to Zipper et al. (2023). All but a few studies were added to the GroMoPo database by the GroMoPo team to kick-start the initiative. In the future, the GroMoPo initiative aims to engage the groundwater modelling community by encouraging authors to upload information themselves.

New database entries can be added via an online form set up within the open-source Streamlit (Streamlit 2025) application (Gromopo 2025 and Zipper et al. 2023). When submitting information about a groundwater model, the new database entry is automatically submitted to the HydroShare data repository (Hydroshare 2025), hosted by CUAHSI (The Consortium of Universities for the Advancement of Hydrologic Sciences), and the GroMoPo database is updated using a tag created in the form submission. Accordingly, the GroMoPo application allows users to browse through the database by selecting groundwater models from a map—see Figure S1 in the electronic supplementary material (ESM)—to visualise the corresponding metadata. There are several metadata attribute themes, ranging from general to technical information (Table 1). Further information and a detailed description of the GroMoPo database can be found in Zipper et al. (2023).

**Table 1:** GroMoPo attribute themes and information collected through an online form

Attribute theme	Information collected
General information	Author names and contact emails^a^ Link to report/article (e.g. DOI)^a^ Publication title, abstract and publication year^a^ Country of primary model developer or institution and whether the model developer’s institute is located in the same country as the model location^a^ Model data availability (e.g. report/paper only, input/output publicly available)^a^
Spatial extent	Bounding box coordinates^a^ Model scale (in km^2^)
Geological information	Dominant geological material in the model domain Geological data availability
Model general information	Number of model layers Temporal extent Maximum model depth
Model technical information	Numerical code used Model purpose (e.g. climate change scenario) Model calibration Model coupling (e.g. to surface-water model)

^a^Indicates a mandatory field

**Fig. 2:**
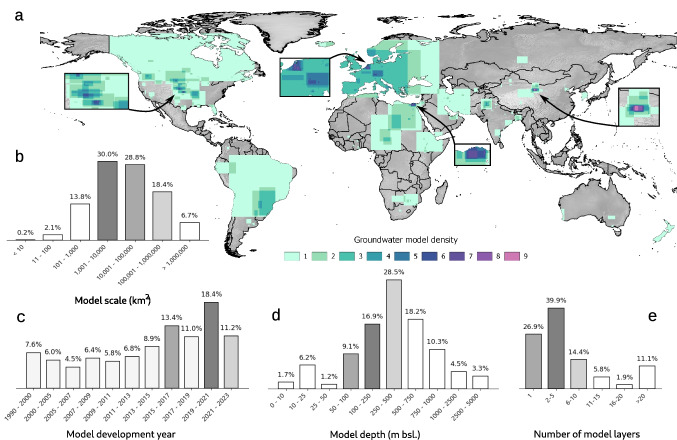
**a** Global coverage of groundwater models and several attributes inside the GroMoPo database.** b** The most common model scale (km^2^) found in groundwater models, **c** the number of groundwater models developed during the last four decades,** d** the most common depth (meters below sea level) considered in groundwater models, and** e** the most common number of groundwater model layers used in groundwater simulations

The main focus of this study is to analyse how common trends and biases in groundwater modelling from recent decades are reflected in the GroMoPo database. Additionally, a conceptualisation of future GroMoPo improvements and goals is presented. To identify trends and biases in the GroMoPo database and in the groundwater community more broadly, a two-fold analysis was conducted. First, model data were aggregated into specific attribute themes (Fig. 2); Table 1 visualises the distribution of the key model attributes. Second, the spatial extent of each groundwater model on GroMoPo is used to correlate model attributes with multiple global datasets to investigate potential biases and trends in the collected groundwater model studies. Socio-economic factors such as gross domestic product (Kummu et al. 2018) (GDP), population density (Center for International Earth Science Information Network - CIESIN - Columbia University 2018) or human development index (Kummu et al. 2018) (HDI) were considered as well as physical factors like observed groundwater-table decline (Kuzma et al. 2023), water-table depth (Fan et al. 2013) (WTD), lithology type (Hartmann and Moosdorf 2012) and climate zone (Beck et al. 2018). The centroid of each model bounding box is used to extract information from global datasets. The spatial distribution of the centroid points overlaid over the global datasets is provided in the ESM (see Figures S3–S5 therein).

## Results

### Geographical, spatial and temporal distribution of groundwater models

**Fig. 3:**
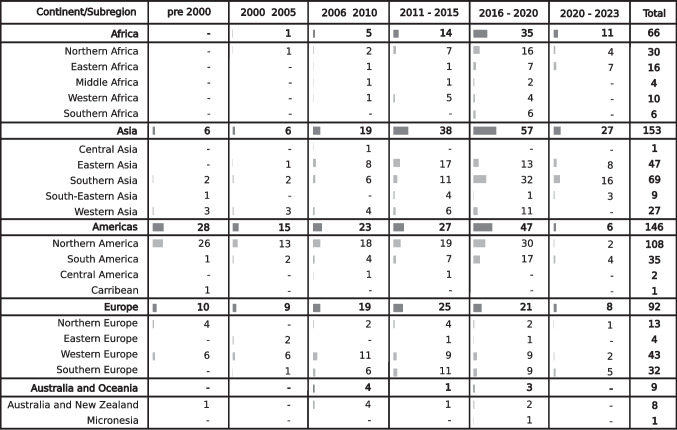
Number of collected groundwater model studies based on their time of publication and geographical location (per continent and United Nations defined subregions). The size bars represent the percentage of studies published in each time period on continental and subregional scale

The geographical extent of the collected groundwater models covers predominantly Europe and North and South America, with relatively lower coverage in Africa, Asia and Australia (Fig. 2a). Only nine models currently in the database are from Australia and Oceania, despite Australia and New Zealand having a strong history and expertise in groundwater modelling. Several regions show a high density of groundwater models (e.g. the Netherlands, the Nile Delta, Northern China and the Midwest of the United States of America). These regions are known for relying on groundwater extraction to sustain the regional freshwater demand; however, other groundwater-dependent regions (e.g. Mekong Delta) facing groundwater depletion are not yet represented in GroMoPo, and further data collection efforts are necessary. Figure 3 also highlights differences among continental subregions (defined by the United Nations)—for instance, in Africa, most groundwater model studies are concentrated in the northern and eastern parts of the continent. This hints at a higher reliance on groundwater resources in these areas, particularly in the Nile Delta region. Similarly, in Asia, most groundwater model studies are found in the eastern (e.g. China, Japan) and southern (e.g. India, Pakistan, Iran) subregions. Several subregions around the world have a conspicuously limited number of groundwater model studies, such as middle, western, and southern Africa; Central and Southeast Asia; Central America and the Caribbean; and Eastern Europe. This scarcity may partly stem from gaps in the GroMoPo data collection efforts, despite a strong focus on identifying groundwater model studies in these areas with poor database coverage; furthermore, it underscores the need for enhanced groundwater modelling education, funding, and applications in these underrepresented subregions.

More than half of the articles and reports collected depict groundwater models covering domains between 1001 and 100,000 km^2^ (see Fig. 2b), a scale that can be considered to characterise local-to-regional groundwater flow patterns (Gleeson and Paszkowski 2014). One-quarter of the collected groundwater models consider areas larger than 100,000 km^2^, thus representing supra-regional, national, and continental-scale models.

There is a clear trend of an increasing number of groundwater models being developed and published since 2010 (Fig. 3). This could be associated with increased access to more powerful computational facilities, increased Web of Science records allowing more models to be discovered, more efficient and accessible numerical codes and (free) software packages, and/or an accelerating necessity to understand current and future groundwater conditions to secure water supply human consumption and ecological sustainability. The growing focus on groundwater modelling research (Jia et al. 2020) in recent years also highlights the importance and utility of a globally centralised database of groundwater model metadata and related studies.

### Knowledge centres and knowledge transfers in groundwater modelling

**Fig. 4:**
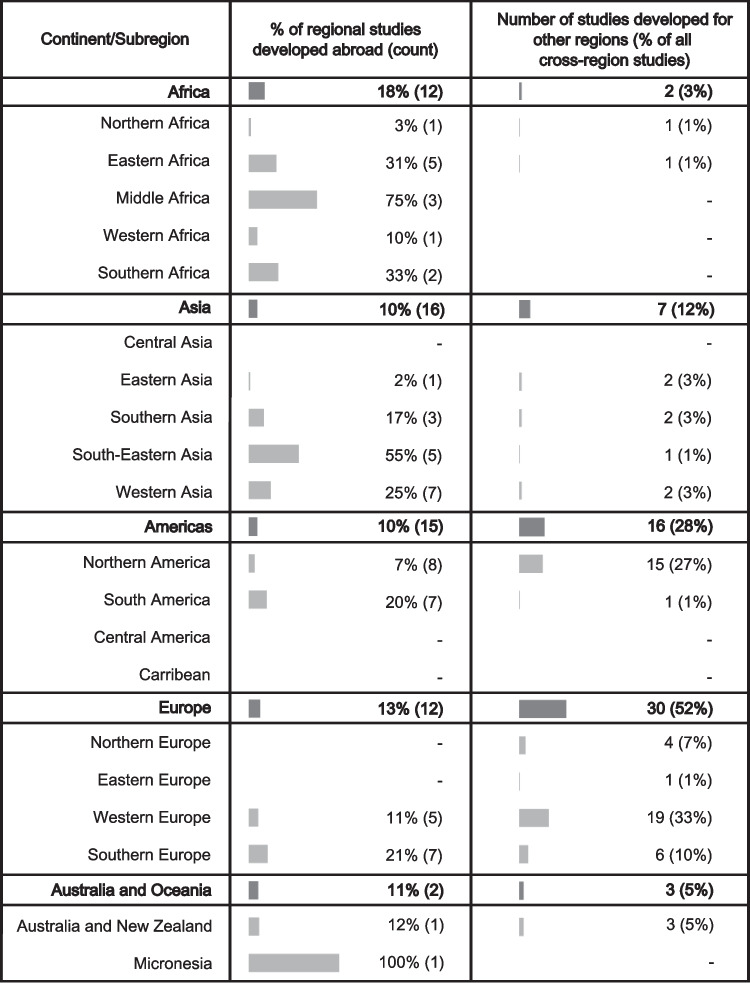
Summary of groundwater modelling knowledge transfers across continents and subregions

The primary centres for groundwater modelling, based on the current GroMoPo database, are in Asia, Europe, and North America (Fig. 3). To assess potential knowledge transfer between different regions, a comparison is drawn between the areas being studied and the countries where the institutions conducting the groundwater model studies are based. The findings presented here reveal that approximately 85% of groundwater model studies compiled in GroMoPo were developed locally (Fig. 4).

Trends emerge when examining the 15% of studies that were developed outside their local regions. The subregions with the highest proportion of nonlocally developed groundwater model studies include eastern, middle, and southern Africa, as well as southeastern and western Asia. These subregions also have the lowest overall number of groundwater model studies, highlighting a significant knowledge gap in groundwater modelling within these areas (Fig. 3).

Unsurprisingly, the major knowledge centres are also responsible for the highest share of cross-regional groundwater modelling studies. Together, Asia, Europe, and North America account for 80% of these cross-regional studies. This trend can likely be attributed to the significant scientific funding available in Europe and North America for research initiatives beyond their borders.

### Socio-economic, environmental and technical patterns in groundwater model development

**Fig. 5:**
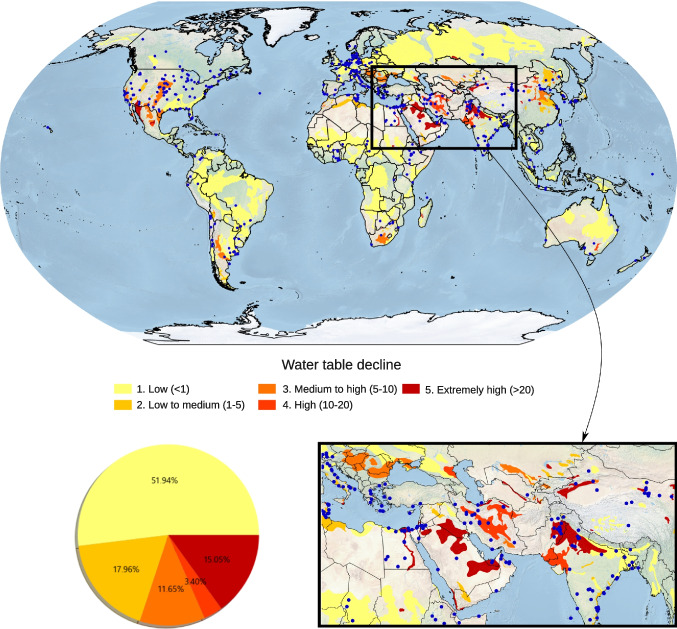
Location of collected groundwater model studies expressed as blue centroid points of the rectangle bounding boxes shown in Fig. 2 overlaid over estimated water-table decline (Kuzma et al. 2023). The pie chart shows the percentage of collected groundwater model studies that fall within each estimated water-table decline range

Groundwater-table decline (Kuzma et al. 2023) and groundwater depletion is a growing issue in many regions worldwide (see Fig. 5). Correspondingly, more than a third of the groundwater model studies on GroMoPo are in regions with medium to extremely high groundwater stress—Kuzma et al. (2023); see Figure S2 in the ESM. The GroMoPo database has a high density of models in regions with the highest groundwater-table decline and groundwater depletion, namely the Midwest and Western USA, the Middle East and the Ganges and Indus basins in the Indian subcontinent, perhaps reflecting a heavier research focus on areas with stressed groundwater resources.

**Fig. 6:**
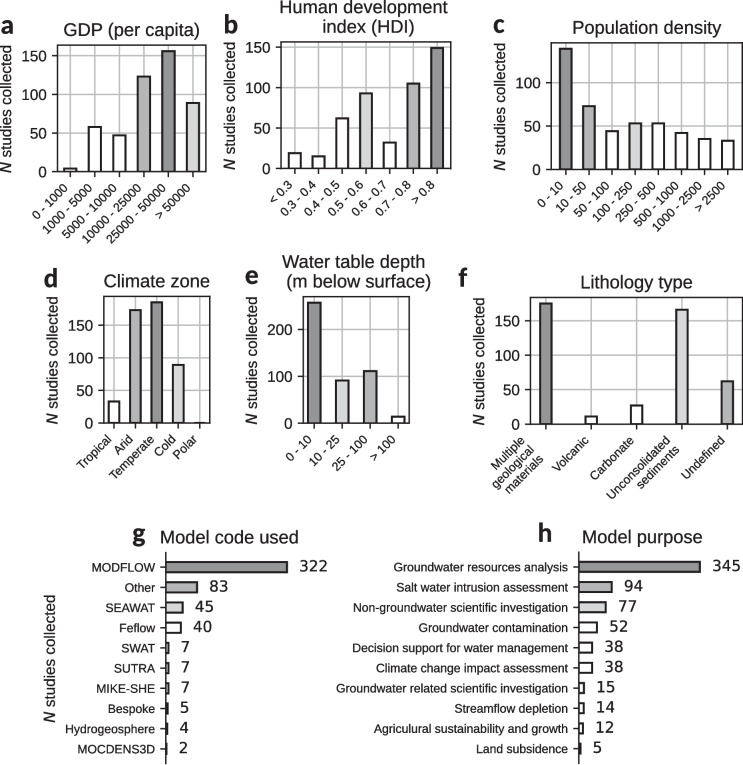
Socio-economic attributes: **a** GDP (Kummu et al. 2018), **b** HDI (Kummu et al. 2018) and **c** population density (Center for International Earth Science Information Network - CIESIN - Columbia University 2018); and physical attributes: **d** climate zone (Beck et al. 2018), **e** water-table depth (Fan et al. 2013) and **f** lithology type (Hartmann and Moosdorf 2012) for collected groundwater model studies. The most used numerical codes (**g**) and model purpose for each study (**h**) are also shown. More than one model purpose can be identified in one study

The analysis presented here also illustrates a socio-economic bias of groundwater models being more likely to be developed for wealthier and more developed regions: most models represent regions with a human development index (HDI) > 0.7 and gross domestic product (GDP) > USD 10,000 per capita based on global gridded HDI and GDP data for the year 2015 from Kummu et al. 2018 (Fig. 6b and S3 of the ESM). The number of groundwater model studies located in regions of Africa and East and Southeast Asia is much lower in general, though there are dense clusters of groundwater modelling studies in specific countries like India, China or Ethiopia. This socio-economic bias is expected since regions like North America and Europe have been at the forefront of groundwater modelling development and research (see Figs. 3 and 4). Additionally, costs associated with groundwater monitoring, groundwater modelling, and investment into (and accessibility of) higher education limit groundwater model development in economically impoverished regions. Access to computational facilities and groundwater modelling software licence costs may also play a role in groundwater models being prevalent in wealthier regions, although open-source and free software is widely available. The costs associated with supporting the time of groundwater modellers, whether academic or in industry, to develop and/or publish the models may be an additional challenge.

Furthermore, the analysis presented here shows that most groundwater models on GroMoPo cover relatively uninhabited regions or regions with quite low population density, especially when looking at groundwater models developed in the USA (Fig. 6c and S5 of the ESM). This bias towards rural regions could be linked to groundwater being mostly used for agriculture in more sparsely populated areas, which would also correspond to the regions with higher groundwater head table decline shown in Fig. 5. The low population density bias might be caused by an averaging effect—when a groundwater model that includes a densely populated city also encompasses surrounding rural areas, the overall average population density in the model domain can appear low (see Fig. 2). However, groundwater models in Asia and Europe represent regions with slightly higher population densities, potentially reflecting greater overall population densities in these regions and/or greater depletion associated with nonagricultural uses.

When looking at the distribution of physical attributes (Fig. 4d–f and S6–S7 of the ESM) of the collected groundwater models studies, it is clear that most groundwater models are in regions with either arid or temperate climate zones with shallow water-table depth and consisting mostly of unconsolidated sediments or multiple geological materials (e.g. mixed lithologies with unconsolidated sediment layers and sedimentary rocks). Groundwater models in regions with volcanic and carbonate systems are under-represented in the current GroMoPo dataset.

The most popular numerical codes applied in groundwater modelling within GroMoPo are the USGS-developed MODFLOW and SEAWAT, being used in more than 80% of the groundwater models collected (Fig. 6g). These numerical codes are open source and therefore provide an excellent opportunity for future input and output groundwater model FAIR data sharing. Amongst other popular numerical codes used in groundwater modelling are the DHI-developed FEFLOW and MIKE-SHE; these, however, are not open source, which might be one reason for their lower usage compared to the USGS-developed numerical codes.

Finally, the analysis presented here shows the main purpose of groundwater modelling studies on GroMoPo is to assess groundwater supply via the availability of groundwater volumes, drawdown levels, etc. (Fig. 6h). Groundwater quality is also a key focus of groundwater modelling studies, with more than 30% of the collected groundwater modelling studies dealing with the effects of saltwater intrusion or groundwater contamination. Other common purposes for groundwater modelling are decision support for water management, general scientific investigations, streamflow depletion, climate change impact assessments, agricultural sustainability and growth, and land subsidence.

## Discussion

The process of collecting groundwater model metadata and information from individual reports and articles is an important step towards a better understanding of the use and trends in groundwater modelling. Furthermore, it helps the groundwater community to assess which systems have been characterised with models that provide opportunities for further study.

While trying to avoid geographical bias through a systematic review approach (see Figs. 2 and 3), there is a visible lack of groundwater model studies collected from several regions. This could be caused by a potential lingering bias (despite best efforts) in collecting the groundwater modelling studies, as is suggested by the limited number of groundwater models in Australia, a region considered to be at the forefront of groundwater modelling research and applications. In other regions, such as parts of Africa and Asia, the lack of groundwater models can be linked to lower access to higher education and groundwater modelling experience. These regions are also more likely to collaborate with foreign partners to build groundwater models and assess groundwater resources, especially from Europe or North America. Furthermore, the search was limited to published studies in English only, while local and regional groundwater modelling reports and scientific articles may be published in other languages in regions such as South America or Africa, or exist outside peer-reviewed journals indexed in the Web of Science. There are certainly additional groundwater model studies from economically developing regions that may be published as technical reports or as master theses. Future work on the GroMoPo database will need to confront this bias and should focus on expanding the GroMoPo community beyond its current North American and European membership. The purpose of this study is to facilitate future engagement with government agencies and other entities with large collections of groundwater models to enable bulk imports of their metadata into GroMoPo, although it can be expected that this could propagate the geographical bias already present in the current GroMoPo dataset.

Another factor behind the selection bias could be the financial and physical requirements for groundwater modelling and groundwater use in general. Monitoring wells and modelling software licences impose a significant financial hurdle to the development of groundwater models, and groundwater modelling may be a nonpriority in governmental spending in impoverished regions. Additionally, groundwater modelling expertise is often limited to a small group of experts, which can lead to limited output of groundwater model studies in these regions, and employing such expertise can lead to groundwater modelling representing a substantial expense for groundwater-related projects. In the future development of GroMoPo, it would be of great benefit and interest to bring in local partners (from academic, governmental or private sector) to help populate the database and gain new insights into the current knowledge gaps in groundwater modelling.

One of the main issues encountered during the data collection process was the delineation of the groundwater model study area. Most studies unfortunately did not provide a shapefile with the exact groundwater model boundary, so it was decided to approximate the area covered by expanding a bounding box over the depicted model boundary in a given study or report (see Fig. 3). In the presented analysis, centroids of these bounding boxes were used to explore the relationship between the groundwater model location and various categories such as HDI, GDP or groundwater table drop (see Fig. 4). This simplification can lead to potential errors in the analysis when extracting values from a global raster dataset (e.g. population density). However, most groundwater models collected in this study so far are small, with around 75% of groundwater models covering less than 100,000 km^2^. Therefore, the analysis by centroid points presumably shows a higher degree of certainty than it would over larger groundwater model scales. In future upgrades, GroMoPo will be able to accommodate additional model domain geometries beyond the square bounding extent, where polygons provided with existing model submissions can be visualised instead of their bounding boxes.

The online Streamlit application (Fig. 2) simplifies the collection of groundwater model metadata, making it more efficient and accessible to the entire hydrogeological community. The ambition for GroMoPo is to become a community standard by providing groundwater model developers with a routine method to publish their metadata alongside their academic articles. Currently, completing the form and submitting groundwater model metadata to GroMoPo takes only a few minutes. This minimal commitment can lead to greater visibility, knowledge exchange, and potential collaborations. In the future, the GroMoPo initiative aims to engage scientific journal editors who could encourage authors to submit their groundwater model metadata to GroMoPo before publishing their articles. Additionally, this study will hopefully lead to future collaborations with other international partners (such as IGRAC) to host the GroMoPo platform and help promote the database amongst the larger hydrological, water management, and policymaking community.

The main goal of GroMoPo is to make groundwater models FAIR and accessible to facilitate future groundwater modelling, management, policy-making, research, and education opportunities. This study illustrated how GroMoPo enables analysis of the groundwater modelling metadata and documentation to identify trends and biases in groundwater flow models globally. Collecting several key metadata attributes for more than 450 groundwater models enabled an analysis of the most common practices and parameters in groundwater modelling. Most of the studies collected come from economically developed regions, highlighting a potential knowledge gap in the current GroMoPo version. GroMoPo can contribute to bridging this gap by providing a platform for the groundwater modelling community to exchange experiences and groundwater modelling knowledge worldwide (and hopefully also input data and code in the future), leading to better interoperability and reusability of groundwater models. The open access to GroMoPo can also lead to interesting insights and analyses of groundwater models by any member of the groundwater modelling community with an interest in exploring the database.

This study aspires to ignite interest in collecting groundwater model knowledge and information in the future, and thus help to gain new insights into groundwater modelling as well as connect the individual members of the groundwater modelling community. The GroMoPo initiative would, therefore, like to appeal to the members of the community and invite them to share groundwater model metadata and information. In this way, the groundwater models will be easier to find, access, and share in the future, promoting the FAIR principle, which is becoming the standard across all scientific fields.

## Supplementary Information

Below is the link to the electronic supplementary material.

Supplementary file1 (PDF 2.32 MB)

## Data Availability

The GroMoPo platform is accessible at https://www.gromopo.org. The complete GroMoPo database is hosted on HydroShare: https://www.hydroshare.org/resource/114b76f89d1c41c38e0e235443c7544c/.

## Code Availability

Python code that is used to set up the GroMoPo platform can be found at https://github.com/Gromopo/GroMoPo.
