# Supplementary material for: Current trends and biases in groundwater modelling using the community-driven groundwater model portal (GroMoPo)
Source: Hydrogeol J. 2025 Mar 28;33(2):355–66. doi: 10.1007/s10040-025-02882-7 (PMC11976367; doi:10.1007/s10040-025-02882-7)
Supplement: Supplementary file 1 — Supplementary file1 (PDF 2.32 MB) [file 10040_2025_2882_MOESM1_ESM.pdf]

Hydrogeology Journal: Electronic supplementary material

Current trends and biases in groundwater modelling using the community-driven groundwater model portal (GroMoPo)

Authors

Daniel Zamrsky[1], Sacha Ruzzante[2], Kyle Compare[3], Daniel Kretschmer[4,5], Sam Zipper[6,7], Kevin M. Befus[8], Robert Reinecke[4,5], Yara Pasner[9] Tom Gleeson[2], Kristen Jordan[6], Mark Cuthbert[10], Anthony M. Castronova[11], Thorsten Wagener[4], Marc F.P. Bierkens[1,12]

Affiliations

1. Department of Physical Geography, Utrecht University, Utrecht, The Netherlands
2. Department of Civil Engineering and School of Earth and Ocean Sciences, University of Victoria, Canada
3. Department of Earth, Ocean & Atmospheric Science, Florida State University, Tallahassee, FL, USA
4. Institute of Environmental Science and Geography, University of Potsdam, Potsdam, Germany
5. Institute of Geography, Johannes Gutenberg-University Mainz, Mainz, Germany
6. Kansas Geological Survey, University of Kansas, Lawrence KS, USA
7. Department of Geology, University of Kansas, Lawrence KS, USA
8. Department of Geosciences, University of Arkansas, Fayetteville, AR USA
9. University of California, Davis, USA
10. School of Earth & Environmental Sciences, Cardiff University, UK
11. Consortium of Universities for the Advancement of Hydrologic Sciences, Inc
12. Deltares, Unit Subsurface and Groundwater Systems, Utrecht, The Netherlands

corresponding author(s): Daniel Zamrsky (d.zamrsky@uu.nl)

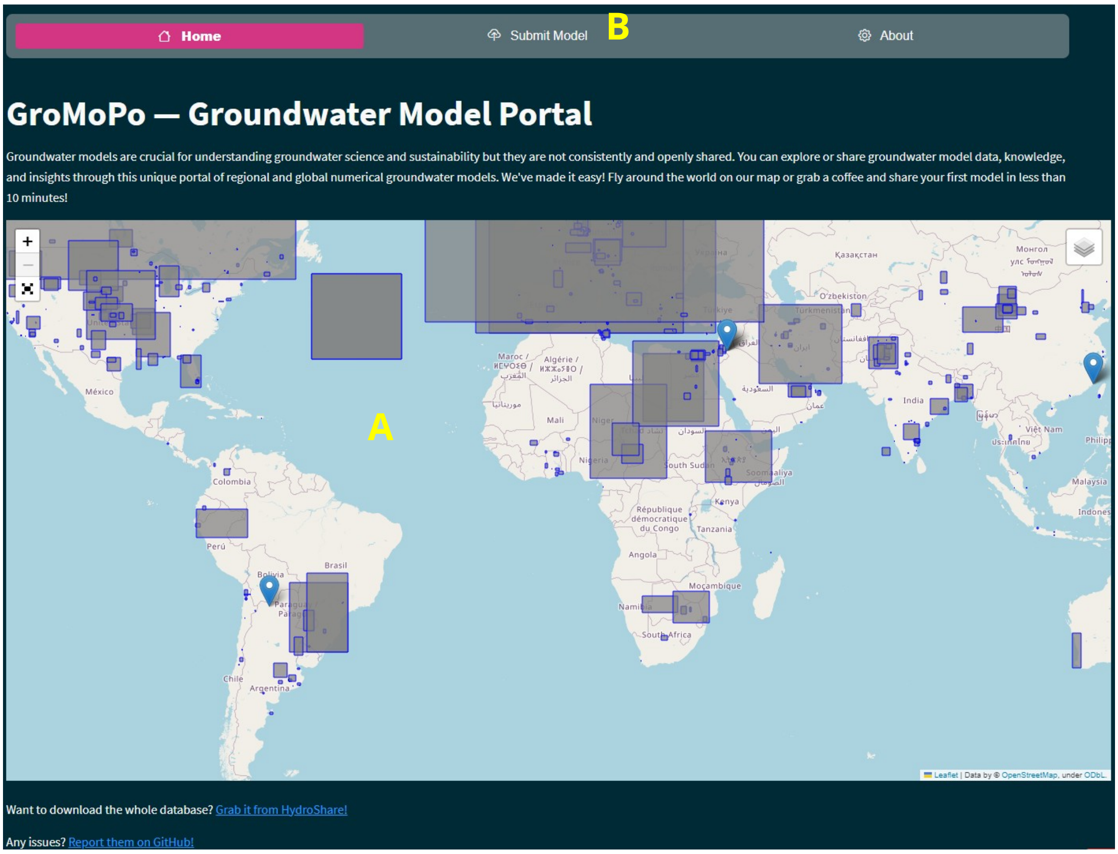

Figure S1 GroMoPo Streamlit application showing the bounding boxes of the collected groundwater model information (A). In the Submit Model tab (B) potential users can submit new groundwater model information.

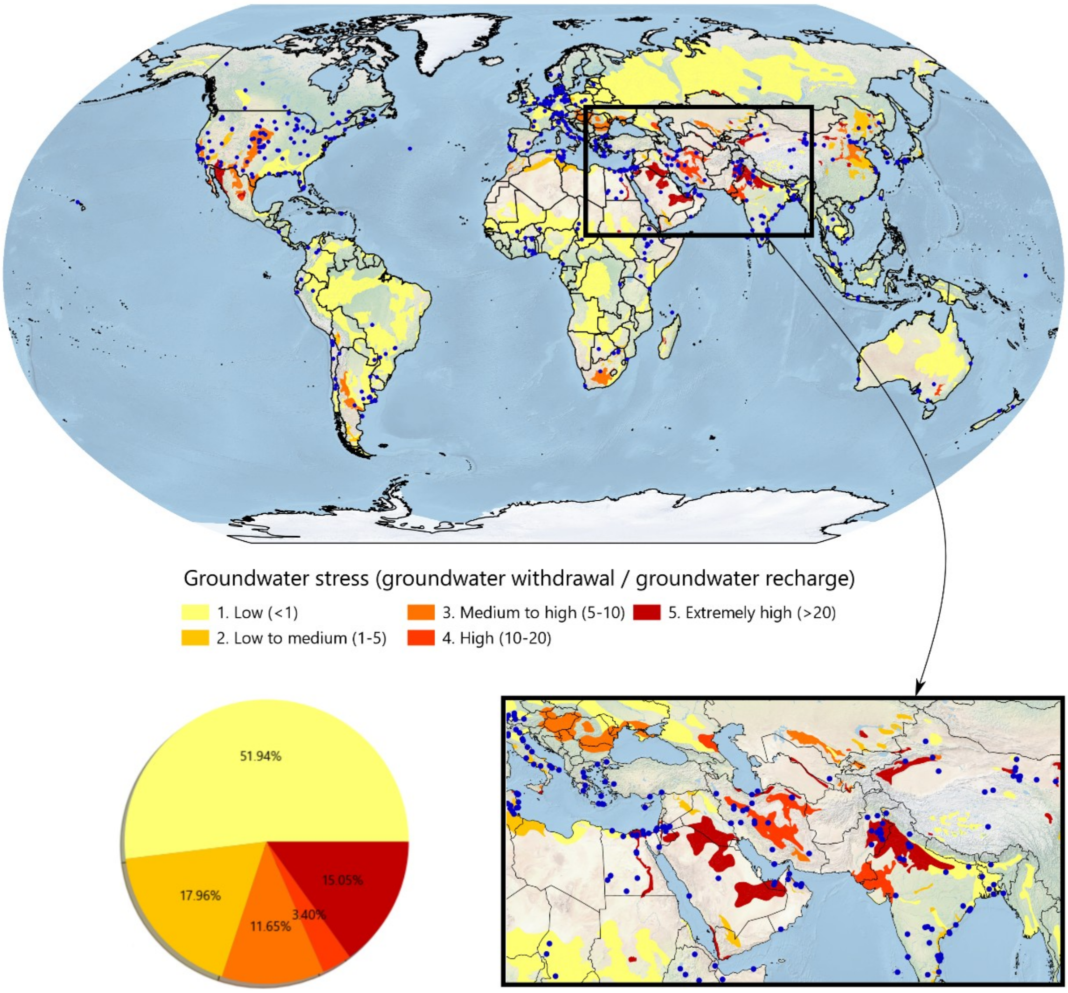

Figure S2 Location of collected groundwater model studies expressed as centroid points of the rectangle bounding boxes shown in Figure 2 overlaid over estimated groundwater stress (Kuzma et al., 2023).

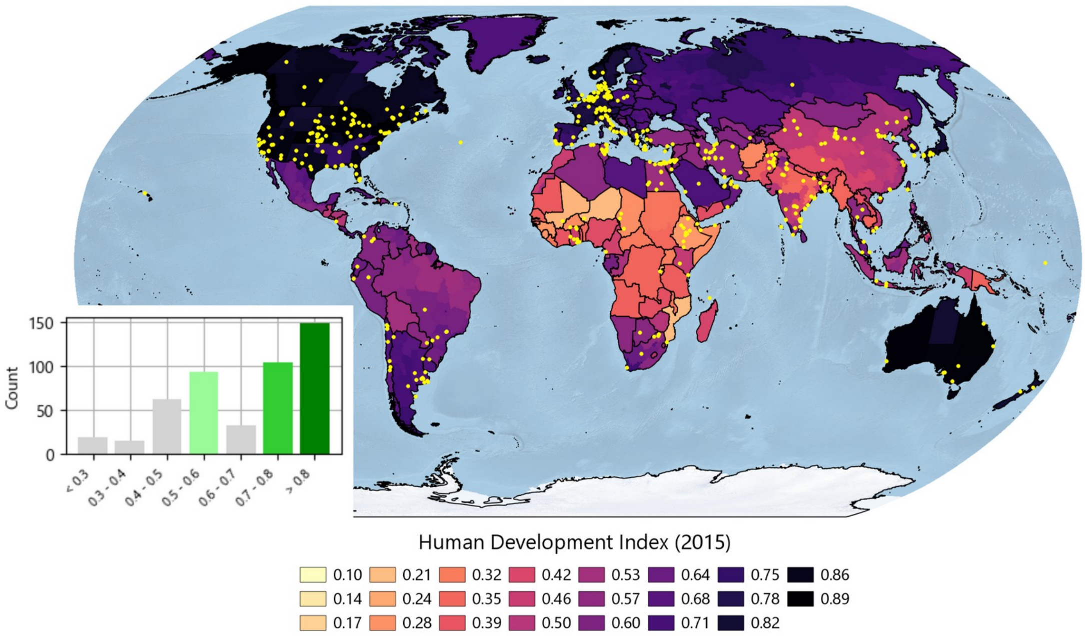

Figure S3 Location of collected groundwater model studies expressed as centroid points of the rectangle bounding boxes shown in Figure 2 overlaid over human development index (HDI) dataset (Kummu et al., 2018).

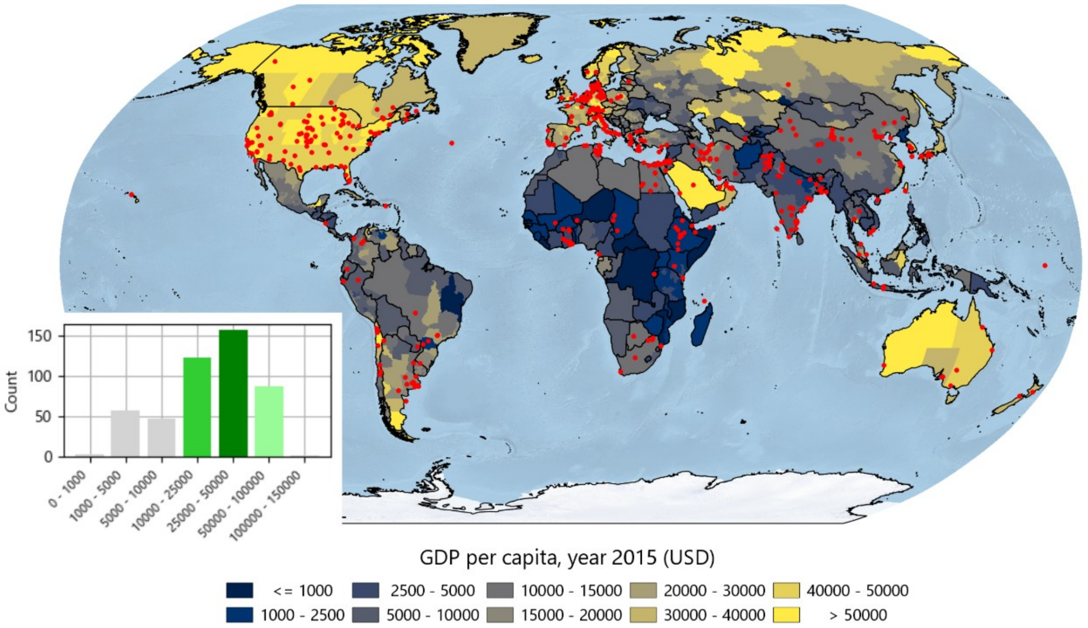

Figure S4 Location of collected groundwater model studies expressed as centroid points of the rectangle bounding boxes shown in Figure 2 overlaid over the gross domestic product (GDP) dataset (Kummu et al., 2018).

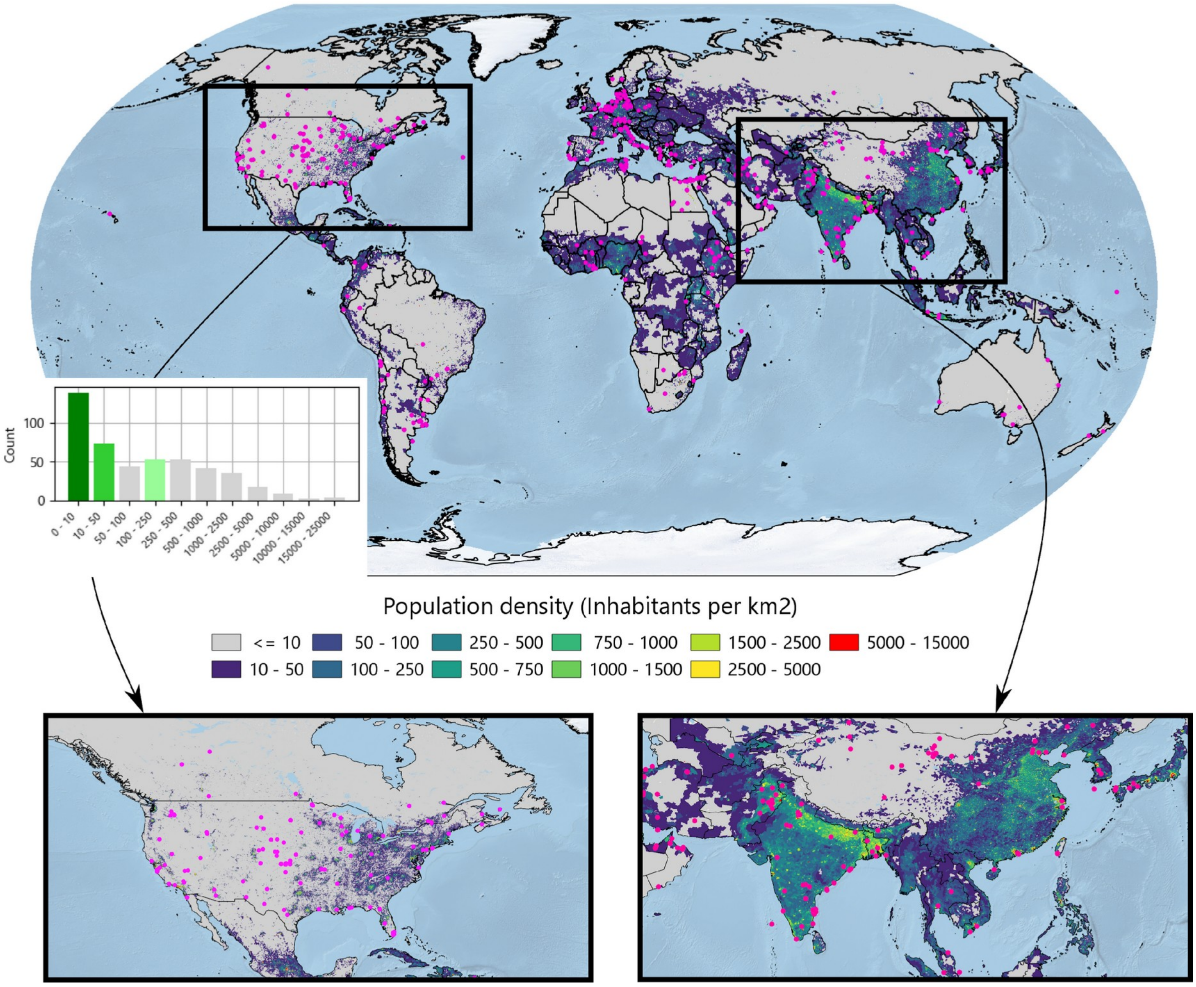

Figure S5 Location of collected groundwater model studies expressed as centroid points of the rectangle bounding boxes shown in Figure 2 overlaid over global population density dataset (Center for International Earth Science Information Network - CIESIN - Columbia University, 2018).

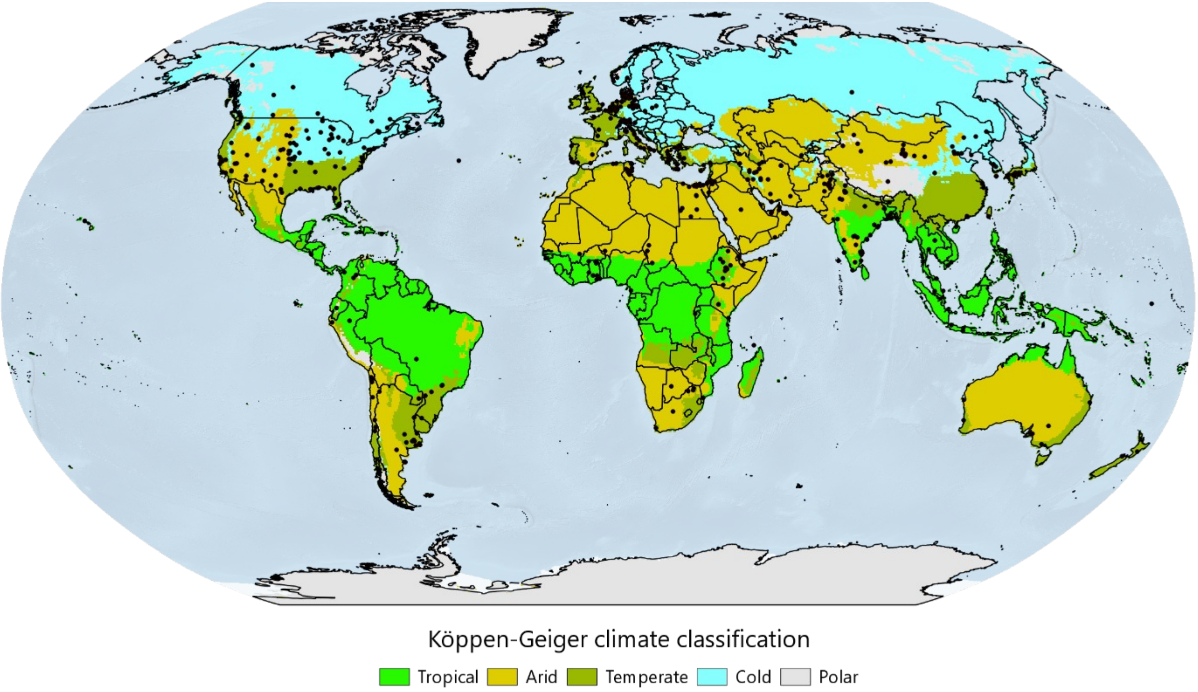

Figure S6 Location of collected groundwater model studies expressed as centroid points of the rectangle bounding boxes shown in Figure 2 overlaid over the Köppen-Geiger climate classification dataset (Beck et al., 2018).

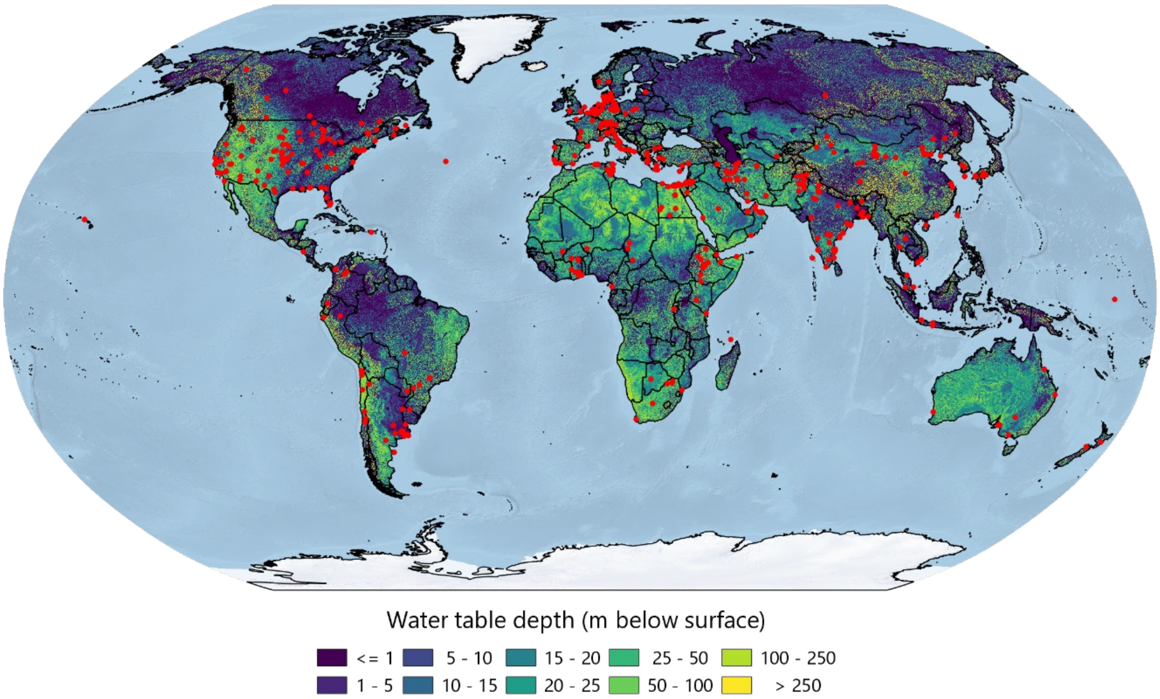

Figure S7 Location of collected groundwater model studies expressed as centroid points of the rectangle bounding boxes shown in Figure 2 overlaid over the global depth to groundwater dataset (Fan et al., 2013).
